# Hemoglobin and transferrin saturation are associated with mobility and physical function two months after hip fracture surgery: an observational cohort study

**DOI:** 10.1007/s41999-026-01407-z

**Published:** 2026-01-16

**Authors:** Martin Aasbrenn, Nicolas Tekin Jones, Camilla Kara Svensson, Marie West Pedersen, Nicolai Henning Jensen, Sune Pedersen, Luana Sandoval Castillo, Thomas Giver Jensen, Troels Haxholdt Lunn, Eckart Pressel, Henrik Palm, Søren Overgaard, Charlotte Suetta, Morten Tange Kristensen

**Affiliations:** 1 Department of Geriatric and Palliative Medicine, Copenhagen University Hospital - Bispebjerg and Frederiksberg, Entrance 60, Ground Floor, Ebba Lunds Vej 44, 2400 Copenhagen, NV Denmark; 2 Department of Clinical Medicine, Faculty of Health and Medical Sciences, University of Copenhagen, Copenhagen, Denmark; 3 Department of Orthopedic Surgery and Traumatology, Copenhagen University Hospital—Bispebjerg and Frederiksberg, Copenhagen, Denmark; 4 Department of Physical and Occupational Therapy, Copenhagen University Hospital—Bispebjerg and Frederiksberg, Copenhagen, Denmark; 5 Department of Geriatric and Palliative Medicine, Copenhagen University Hospital—Amager and Hvidovre, Copenhagen, Denmark; 6 Department of Anaesthesia and Intensive Care, Copenhagen University Hospital—Bispebjerg and Frederiksberg, Copenhagen, Denmark

**Keywords:** Orthogeriatrics, Hip fracture, Anemia, Iron, Mobility

## Abstract

**Purpose:**

To investigate whether hemoglobin two days after discharge and transferrin saturation two months after discharge for hip fracture (HF) were associated with mobility and functional outcomes.

**Methods:**

We included patients ≥ 65 years, surgically treated for a hip fracture at Copenhagen University Hospital, Bispebjerg and Frederiksberg. Iron deficiency was defined as transferrin saturation (TSAT) below 20%. Mobility and physical function were measured two months after discharge in an orthogeriatric outpatient clinic using new mobility score (0–9 points, 9 best functional mobility), six-minute walk test, usual walking speed (10-m), 30-s Sit-to-Stand test, and handgrip strength. Associations between hemoglobin, transferrin saturation (per units of 10%) and outcomes were evaluated by multivariable linear regression, with age, sex, and type of fracture (intra- versus extracapsular) as covariates.

**Results:**

235 patients were included (69% women, age 80 ± 8 years, 55% had intra-capsular fractures). The average ± SD hemoglobin two days after discharge was 10.4 ± 1.5 g/dL, 91% had anemia according to the WHO definition. The average transferrin saturation two months after surgery was 0.21 ± 0.09, 51% had iron deficiency.

High hemoglobin was associated with high new mobility score (*B* = 0.46, 95% CI 0.25–0.66, *p* < 0.001). High transferrin saturation was associated with high new mobility score (*B* = 0.74, 95% CI 0.17–1.31, *p* = 0.01), six-minute walk test (*B* = 47, 95% CI 16–78, *p* = 0.004), walking speed (*B* = 0.12, 95% CI 0.06–0.19, *p* < 0.001), 30-s Sit-to-Stand test (*B* = 1.56, 95% CI 0.30–2.85, *p* = 0.01), and hand grip strength (*B* = 2.1, 95% CI 0.0–4.2, *p* = 0.049).

**Conclusions:**

High hemoglobin two days after discharge was associated with high new mobility score two months after surgery. Transferrin saturation was associated with mobility and all measured functional outcomes two months after hip fractures.

## Key summary points

**Aim:**

We investigated whether anemia and transferrin saturation were associated with mobility and physical function after hip fracture surgery.

**Findings:**

Higher hemoglobin two days after discharge was associated with better mobility two months after surgery for a hip fracture. A higher transferrin saturation two months after surgery was associated with better results at all functional tests and higher mobility.

**Message:**

Targeted intervention studies should be performed to assess whether increased iron availability in the post-operative period could improve long-term mobility and physical function.

## Introduction

Globally, the number of patients above 55 years of age treated for a hip fracture was reported to be 16.8 million in 2019 and projections estimate a 1.9-fold increase by 2050 [1–3]. Patients with hip fractures have a 30-day mortality rate around 10%, a short-term readmission rate at 16% and a significant need for domestic care following discharge [3–6]. Moreover, patients with hip fractures face difficulties regaining their mobility, and approximately 50% have not regained pre-fracture mobility level after one year [7]. Low mobility is associated with higher infection rates, hospital readmissions and increased mortality rates [8–12]. Thus, there is still potential for improving treatments. Some risk factors for lack of regaining mobility have been identified, such as the amount of physical activity during hospitalization, pain, type of surgery, post-operative infections, delirium, and anemia [13].

Anemia is present in a majority of patients after hip fractures [14] and associated with increased mortality [15, 16]. Anemia and iron deficiency can result in limited transport of oxygen to the muscles and reductions in muscle function. Reductions in muscle function can be very serious for frail patients, who may permanently lose the ability to move independently. According to the patients, stable mobility with capability of handling daily activities really matters after a hip fracture [17]. There appears to be an association between anemia on the post-operative days 1–3 and lower mobility [13], but knowledge of the longer-term associations are limited.

Thus, the primary aim of this study was to investigate the association between hemoglobin two days after discharge from hospital and the mobility, evaluated with the New Mobility Score (NMS), two months after discharge for a surgically treated hip fracture. The secondary aims were to evaluate if iron deficiency two months after surgery was associated with mobility and physical function.

## Methods

### Study design

This study was an observational cohort study of patients surgically treated for an acute hip fracture at the Copenhagen University Hospital, Bispebjerg and Frederiksberg (BFH), and seen at the hospital’s orthogeriatric outpatient clinic, during two periods. In the first period, all patients treated for hip fracture from January 1 through December 31, 2021, and seen at the outpatient clinic between February 24, 2021 and March 30, 2022 were included. In 2022 and 2023, an expanded version of blood tests and function tests were performed in the orthogeriatric outpatient clinic. In the second period, patients surgically treated for hip fracture between August 10, 2022 and May 24, 2023 and seen at the outpatient clinic between October 15, 2022 and August 15, 2023 were included.

Inclusion criteria for the orthogeriatric outpatient clinic in both time periods included a low-energy hip fracture, and being able to be transported to the clinic, walk with assistance, and being motivated for further follow-ups. Patients with terminal disease and patients who stayed permanently in bed at the time of follow-up were excluded. Reporting of the study follows the STROBE guideline for observational studies [18].

### Setting and enhanced recovery program

Patients were treated at the acute orthogeriatric hip fracture unit at BFH and followed an enhanced recovery program with early mobilization and in-hospital physiotherapy, followed by the usual municipality-based rehabilitation program. During the admission, there was close collaboration between orthopedic surgeons, geriatricians and anesthesiologists [4] and we aimed to perform surgery within 24 h after admission [19]. The patients were discharged to their own home or a 24-h temporary residential center in the municipality according to their individual needs. All patients were offered two follow-up visits by an orthogeriatric nurse in their residence around 2 and 9 days after discharge [20]. Oral iron supplements, intravenous iron, and tranexamic acid were not administered routinely to patients included in this study. There was no uniform policy about iron treatments at BFH in the time period when this study was conducted. National Danish recommendations for blood management were generally followed but could be overruled at the discretion of the physician.

### Study visits

Data were registered at five time points: day of admission to hospital (T1), discharge from hospital (T2), first home visit (T3, 2 days after discharge), second home visit (T4, 9 days after discharge), and outpatient visit (T5, about two months after discharge).

### Outcomes and variables

#### Primary outcome

The primary outcome was NMS registered at the outpatient visit (T5). NMS evaluates the gait function indoor, outdoor and during shopping, and is a validated score for orthogeriatric patients. The total NMS score ranges from 0 to 9, where 9 represents the highest functional mobility [21, 22].

#### Other variables

In the first inclusion period in 2021, the included variables were age, sex, type of hip fracture, NMS (T2 and T5), Cumulated Ambulation Score (CAS) (T2 and T5), 30-s-sit-to-stand-test (30 s-STS) (T5), and hemoglobin (T1, T2, T3, T4, and T5). In the second period from 2022 to 2023, six-minute walk test (6MWT), 10-m walking speed, hand grip strength, transferrin saturation, and ferritin (T5) were added.

#### Definition of anemia

Anemia was defined according to the WHO definition as present if below 12 g/dL in woman and below 13 g/dL in men [23].

#### Definition of iron deficiency

Iron deficiency was defined as a transferrin saturation below 20% [24].

#### Functional evaluation

CAS is a validated score to evaluate basic mobility in orthogeriatric patients and other patient groups. CAS ranges from 0 to 6, higher values indicate better ambulatory capacity. Higher scores indicate more independent gait function. The 30 s-STS (number of repetitions in 30 s), hand-grip strength (best of 3 assessments with dominant hand), walking speed (10 m with usual speed), and 6MWT (meters walked in 6 min) were done according to standardized manuals [25–28]. All functional tests were performed by experienced physiotherapists in the outpatient clinic.

### Statistical methods

Continuous data are presented as mean (standard deviation (SD)) and median (IQR, range) for variables that do not follow the normal distribution. The associations of hemoglobin and transferrin saturation (per units of 10%) with NMS and physical function were evaluated by multivariable linear regression analysis with age, sex, and type of hip fracture (intra- versus extracapsular) as covariates. Unadjusted and adjusted results are presented with 95% confidence intervals. *P* values < 0.05 were considered statistically significant.

## Results

**Fig. 1:**
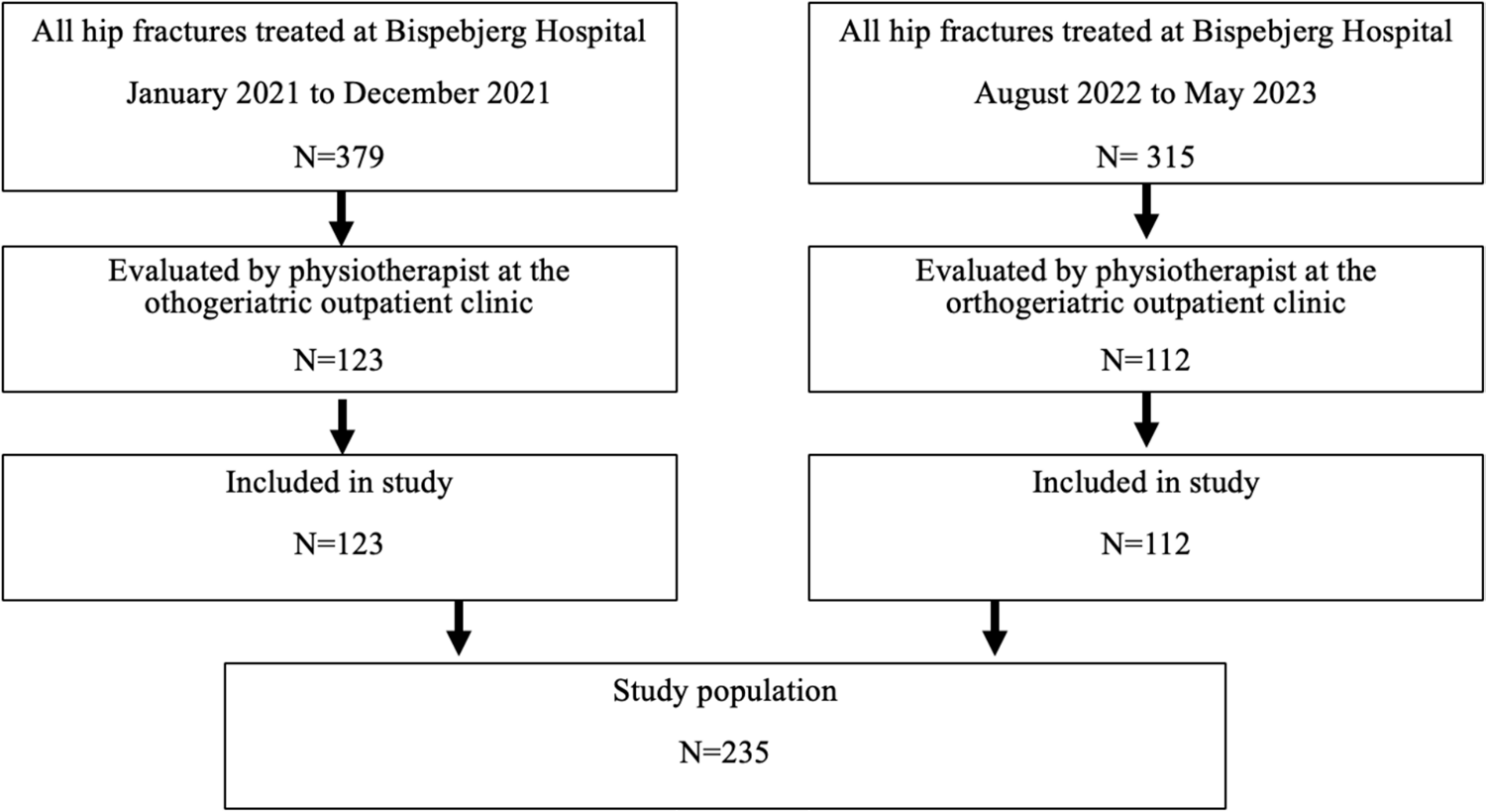
Inclusion of orthogeriatric patients in two separate time periods

In total, 235 patients (69% women) with a mean age of 79 years were included, 55% had intra-capsular fractures. Figure 1 depicts the inclusion of patients.

**Fig. 2:**
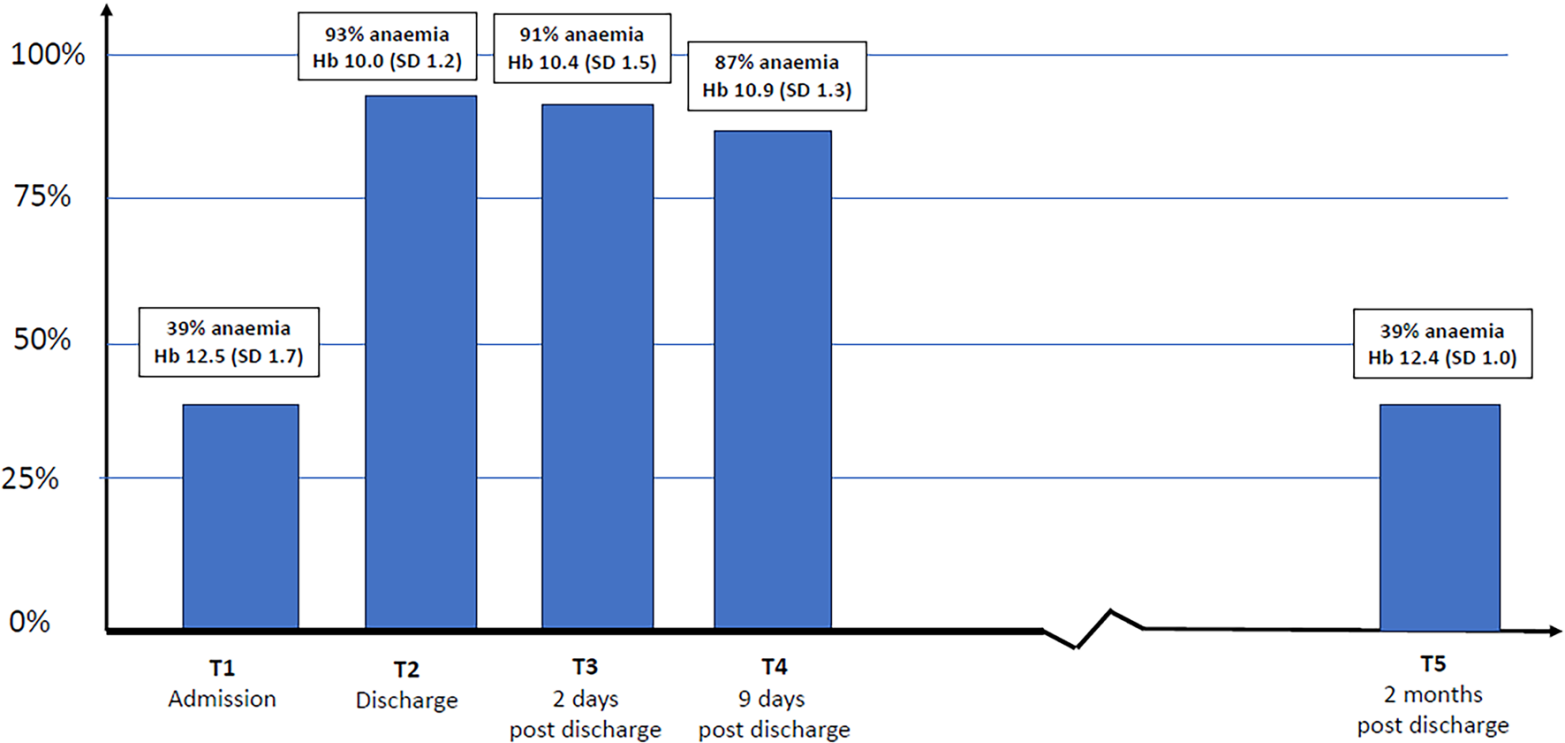
Prevalence of anemia and hemoglobin at five time points

The prevalence of anemia was around 90% at three time points around discharge and fell to about 40% at two months follow-up (Fig. 2). Two months after discharge following surgery for hip fracture, iron deficiency was present in 51% of the patients (Table 1).

**Table 1:** Patient characteristics at inclusion and iron status two months after discharge

	All *N* = 235	Period 1 (2021) *N* = 123	Period 2 (2022–2023) *N* = 112
Age (years)	79.5 (8.3)	78.6 (8.3)	80.6 (8.2)
Sex (% women)	161/235 (69%)	88/123 (71%)	73/112 (65%)
Intracapsular fractures	129/235 (55%)	65/123 (53%)	64/112 (57%)
Iron deficiency (T5)			40/78 (51%)
Transferrin saturation (T5)			0.21 (0.09)
Ferritin (µg/l) (T5)			112 (76–193, 19–2380)

Data given as ratio (percentage), mean (standard deviation) or median (interquartile range, range)

*T5* outpatient visit, two months after discharge

Almost all patients were independent in basic mobility (CAS = 6 points) at the 2-month visit, while the NMS on average was 4.9 (SD 2.5). Patients walked on average 253 m in the 6MWT, and used 15.3 s to walk ten meters, corresponding to an average walking speed of 0.79 m/s (SD 0.29) (Table 2).

**Table 2:** Mobility and physical functional ability at the two months follow-up

	All *N* = 235	Period 1 (2021) *N* = 123		Period 2 (2022–2023) *N* = 112	
		Value	*n*	Value	*N*
New mobility score (NMS, 0–9 points)	4.9 (2.5)	4.7 (2.2)	123	5.1 (2.8)	112
Independent Cumulated Ambulation Score (6 points)	210/235 (89%)	115/123 (94%)	123	95/110 (86%)	110
30 s Sit-to-stand-test (repetitions)	5.2 (5.4, 0–26)	4.9 (5.2, 0–16)	103	5.6 (5.6, 0–26)	110
Six-minute walk test, meters)				253 (126, 10–570)	95
Walking speed (meters/s)				0.79 (0.29, 0.17–1.47)	101
Hand grip strength, kg					
Women, *n* = 70				18.9 (5.3, 7.1–33.0)	70
Men, *n* = 37				31.4 (9.5, 13.7–52.0)	37

Data given as ratio (percentage), or mean (standard deviation and range)

Higher hemoglobin two days after discharge (T3) was associated with higher NMS scores two months after discharge, with an unadjusted coefficient *B* = 0.61, 95% CI 0.40–0.82, *p* < 0.001. Adjusted for age, sex and type of fracture an increase in hemoglobin of 1 g/dL was associated with an increase of 0.46 points on the NMS scale (*B* = 0.46, 95% CI 0.25–0.66, *p* < 0.001).

Concerning iron stores, a higher transferrin saturation at the two-month follow-up (T5) was also significantly associated with a higher NMS. An increase of 10% in transferrin saturation was associated with an increase of 0.74 points on the NMS scale (Table 3). Additionally, a high transferrin saturation was associated with better performances of almost all measurements of physical function: 30STS, 6MWT, walking speed, and hand grip strength. For hand grip strength, we saw a difference according to sex, with a stronger association in men (Table 3).

**Table 3:** Multivariable linear regression analysis of the association between transferrin saturation at 2-month follow-up and various functional assessments at 2-month follow-up

	Unadjusted		Adjusted (age, sex and type of fracture)		
	*B* value (95% CI)	*p* values	*B* value (95% CI)	*p* values	*N*
New mobility score (NMS)	0.89 (0.25–1.54)	0.007	0.74 (0.17–1.31)	0.01	77
30 s Sit-to-stand-test, repetitions (30STS)	1.71 (0.37–3.05)	0.01	1.56 (0.30–2.85)	0.02	76
Six-minute walk test (meters) (6MWT)	60 (27–93)	< 0.001	47 (16–78)	0.004	66
Walking speed (m/s)	0.14 (0.08–0.21)	< 0.001	0.12 (0.06–0.19)	< 0.001	71
Hand grip strength (all patients)	2.5 (0.2–4.7)	0.03	2.1(0.0–4.2)	0.049	73
Hand grip strength (women)	0.6 (− 1.1–2.2)	0.51	0.4 (− 1.1–1.9)	0.60	48
Hand grip strength (men)	6.2 (2.7–9.8)	0.001	5.9 (2.3–9.4)	0.002	24

Fracture type categorized as intra-capsular or extracapsular. Analyses with linear regression, B values given per units of 10% transferrin saturation

## Discussion

We studied associations between hemoglobin, transferrin saturation and measures of mobility and physical function in the period after hip fracture surgery. Higher hemoglobin values in the week after surgery were associated with better mobility at 2-month follow-up. A higher transferrin saturation two months after hip fracture was associated with better performances on all measures of mobility and physical function.

The findings of the present study add to the literature that low hemoglobin in the weeks after surgery for hip fracture is associated with impaired patient mobility not just in the days after surgery [13], but also on a longer term. This underlines the importance of assessing hemoglobin, not only immediately after surgery, but also at later time points following surgery for hip fracture. Hemoglobin transports oxygen, and reduced oxygen delivery to the skeletal and cardiac muscles can be a limiting factor in rehabilitation. In a recent feasibility trial, intensified acute in-hospital physiotherapy improved basic mobility, but the main reason for not completing physiotherapy was fatigue [29]. Fatigue is a very common symptom of anemia in old patients [24], and may be an important link between hemoglobin and mobility. If patients with anemia use their muscles less due to fatigue in the weeks after hip fracture, it can have consequences for their mobility level two months after surgery.

It is very difficult to assess iron stores biochemically during a hospital admission for hip fracture as the inflammation due to the fracture and the surgery affects the blood tests used to test for iron deficiency [24]. Therefore, we chose to assess transferrin saturation two months after hip fracture surgery, when the inflammatory load is back to normal. Low transferrin saturation was significantly associated with low mobility and low performance on all functional tests. These findings can be explained by known pathophysiological mechanisms: Iron deficiency can reduce physical work capacity partly due to changes in the morphology, number and activity of mitochondria [30, 31]. As for hemoglobin, assessment for iron deficiency should possibly be considered more often at later time points after surgery for hip fracture. Frail patients with a limited premorbid function are at risk for loss of independence after surgery, and a moderate reduction in physical work capacity can therefore have a huge impact [32]. Iron deficiency can lead to fatigue both through anemia and independently of anemia [24].

In the first weeks after discharge, around 90% of the patients in our study had anemia according to the WHO definition. Most patients with hip fracture get intravenous fluid treatment, and this affects the hemoglobin values, but as shown in the present study, the patients stay anemic for weeks following surgery. This indicates that the high percentage of anemia not only is caused by in-hospital ‘dilution’ due to IV fluids. The cause of post-operative anemia in this patient group might be a combination of three factors: first a predisposition to anemia due to high age and comorbidity, second blood loss from fracture and surgery, and third functional iron deficiency due to the inflammatory load [33–35]. Notably, two months following surgery, we observed a rebound of hemoglobin levels back into the normal range so that 60% of the patients were without anemia.

We wonder whether an accelerated increase in hemoglobin could improve mobility for patients with HF in the months after surgery. This could for instance be done by protecting the patient from further blood loss from fracture or surgery, by blood transfusions or by administration of iron. To protect from further blood loss, there is moderate grade evidence for perioperative tranexamic acid for these patients, with no significant added side effects [36]. Large studies indicate that restrictive blood transfusion is safe [37], but arguably some subgroups might benefit from more liberal blood transfusion [38]. Concerning intravenous iron after HF, the current evidence comes from quite few small studies, but some of them show intriguing results [36, 39]. Selected interventions to correct iron deficiency or to increase hemoglobin in the post-operative phase could be tested in further clinical studies.

The patients’ mobility was assessed both with NMS and CAS. We selected NMS as our primary outcome because we expected a ceiling effect of CAS, where most of the patients would reach the maximum score at last follow-up. This was indeed the case as a large majority of the included patients had the maximum score of 6 on CAS two months after surgery. In comparison, the average NMS was around 5 out of 9 maximum points, indicating that NMS is a more sensitive and suitable measurement instrument for studies of longer-term mobility recovery in this patient group.

The strengths of the study include complete registration of NMS and a comprehensive functional measurement by trained physiotherapists, and inclusion of almost all patients seen in the orthogeriatric outpatient clinic.

A main limitation is that the study is an observational study. A pathophysiological relation between anemia, iron and mobility is plausible, but causality cannot be confirmed by observation studies. The inclusion periods were based on the availability of blood tests in years where the test routines in the outpatient clinic changed several times. Therefore, the part of the cohort where iron deficiency was assessed was of limited size. However, hemoglobin and NMS were evaluated in the full cohort without missing data and the included patients in the two periods had similar characteristics. Another limitation is that the functional assessment can be influenced by i.e., pain, frailty, and comorbidities, and data on these aspects were not available in the current cohort. Patients with high frailty or certain serious comorbidities, such as cardiac failure and chronic kidney disease, may both have a higher risk of anemia and a higher risk of mobility loss following a hip fracture which may bias the results. The current study population is probably not representative of all patients with hip fractures as the functional measurements only were performed in patients seen at an outpatient clinic, which required the patients to be ambulatory and motivated for follow-up. Nursing home residents and patients with severe dementia were therefore underrepresented. The patients included are most likely healthier than the average hip fracture patient. Treatment of anemia has been reported to have a higher impact in patients from nursing homes. It is possible that iron deficiency also has a different impact in patients who live in nursing homes or patients with dementia, groups know to be at high risk for adverse outcomes after hip fracture . More research on these subgroups would be appropriate. The patients in the current study are likely to represent the part of the population with hip fracture that is ambulatory before the hip fracture and has the longest remaining life expectancy after the hip fracture, and who therefore can benefit most from interventions that may improve mobility and physical function.

## Conclusions

High hemoglobin two days after discharge following surgery for hip fracture was associated with better mobility two months after discharge. High transferrin saturation was associated with mobility and all measured functional outcomes two months after discharge.

This calls for future studies investigating whether clinical interventions that increase hemoglobin and iron availability after hip fracture can enhance long-term functional and mobility recovery.
